# A common pathogen in an uncommon site: coronary artery stent meticillin-resistant *Staphylococcus aureus* infection

**DOI:** 10.1099/jmmcr.0.005110

**Published:** 2017-09-25

**Authors:** Krystle Shafer, Catalin Toma, Alison Galdys

**Affiliations:** ^1^​ Critical Care Medicine, University of Pittsburgh Medical Center, Suite 1215, Kaufmann Building, 3471 Fifth Avenue, Pittsburgh, PA 15213, USA; ^2^​ Interventional Cardiology, University of Pittsburgh Medical Center, 200 Lothrop Street, Pittsburgh, PA 15213, USA; ^3^​ IDIM Division, MMC 250, University of Minnesota, 420 Delaware Street SE, Minneapolis, MN 55455, USA

**Keywords:** coronary stent infection, combination therapy, suppressive therapy, coronary stent removal, *Staphylococcus* bacteremia

## Abstract

**Introduction.** Coronary artery stents are an uncommon site for infection. Only a handful of case reports describe this condition, and *Staphylococcus aureus* is the most frequent pathogen. Although rare, coronary stent infections are associated with a high mortality rate.

**Case presentation.** We describe the case of a 50-year-old man with a past medical history of seven prior meticillin-resistant *S. aureus* (MRSA) infections over the previous 12 months, who presented with fever and was found to have persistent MRSA bacteraemia. During his hospital course, he developed chest pain and underwent coronary angiography, which revealed a left circumflex coronary to left atrium fistula, presumably due to endarteritis/sent infection. He was treated with combination parenteral antibiotics that were succeeded by oral suppressive therapy. Six months after his diagnosis of coronary stent infection, he suffered a fatal cardiac arrest.

**Conclusion.** Coronary artery stents are an infrequent source of infection; when they occur, they are typically due to *S. aureus*, have a high mortality and ideally are treated with surgical intervention.

## Abbreviations

CAD, coronary artery disease; HD, hospital day; MRSA, meticillin-resistant Staphylococcus aureus; PCI, percutaneous coronary intervention; TDC, tunneled dialysis catheter.

## Introduction

The World Health Organization cites cardiovascular disease as the leading cause of mortality worldwide, and the majority of these deaths are due to coronary artery disease (CAD). Percutaneous coronary intervention (PCI) and coronary stent placement are among the commonest interventions for the management of CAD, and over a half million hospitalizations in the USA each year involve coronary artery stent placement [1]. Despite the ubiquity of coronary artery stents, infection due to these devices is described in only a handful of case reports. Coronary stent infections are challenging to diagnose and treat, and are associated with a high mortality rate. We describe a case of a coronary artery stent infection that highlights the complexities involved with diagnosing and managing these conditions.

## Case report

A 50-year-old white male presented to a community hospital with a chief complaint of fevers for the past 2 days and was found to have meticillin-resistant *Staphylococcus aureus* (MRSA) bacteraemia. His medical history was notable for multiple medical problems: end-stage renal disease requiring haemodialysis; CAD requiring multiple PCIs, including placement of six drug-eluting stents 1 month prior; diabetes mellitus; and peripheral vascular disease with a right below the knee amputation. The PCI a month prior was done for a left circumflex chronic total occlusion using a retrograde approach via an occluded saphenous vein graft, with stenting from the left main to the mid circumflex. He also had five prior MRSA infections in the previous year (in chronological order): a left foot soft tissue infection; an arteriovenous fistula graft infection requiring explantation of the graft and insertion of a tunnelled dialysis catheter (TDC); an automatic implantable cardioverter-defibrillator (AICD) infection requiring permanent removal of the AICD; and two TDC infections, for each of which the TDC was removed and replaced after line holidays.

## Investigations

At the time of his presentation, the patient was febrile at 38.7 ° C, his heart rate was 103 beats min^−1^, his blood pressure was 134/59 mmHg, with no murmur and no reported stigmata of endocarditis on physical examination. His white blood cell count was 7.4×10^3^ cells µl^− 1^ with no bands and with 87 % neutrophils. His TDC was without purulence and there was no evidence of soft tissue infection. Computed tomography of his abdomen and pelvis revealed no lymphadenopathy, no fluid collection or abscess, and no periosteal reaction or lytic lesions. Blood cultures were positive for *S. aureus*, with an oxacillin MIC of >2 µg ml^−1^ and a vancomycin MIC of 1.5 µg ml^−1^. His initial antibiotic regimen was vancomycin monotherapy, 1000 mg dosed after hemodialysis, and this was switched to daptomycin, dosed 6 mg kg^−1^ every 48 h (given after hemodialysis on dialysis days) on hospital day (HD) 4 due to persistent fevers. His TDC was removed on HD 6 and 2 days later a temporary dialysis catheter was placed, but bacteraemia persisted despite this intervention. On HD 10, a transoesophageal echocardiogram was performed and revealed a native mitral valve mass suggestive of a vegetation that was new as compared to his prior echocardiograms, prompting the addition of gentamicin (initial dose of 3 mg kg^−1^ with repeat dosing dependent on drug levels) to his antibiotic regimen, and on HD 14 rifampin (300 mg every 8 h) was added. On HD 16, he developed chest pain with precordial lead electrocardiogram (ECG) changes and was transferred to a tertiary facility for coronary angiography. Following transfer, daptomycin was changed to vancomycin, and the gentamicin and rifampin were continued.

## Diagnosis

**Fig. 1.**
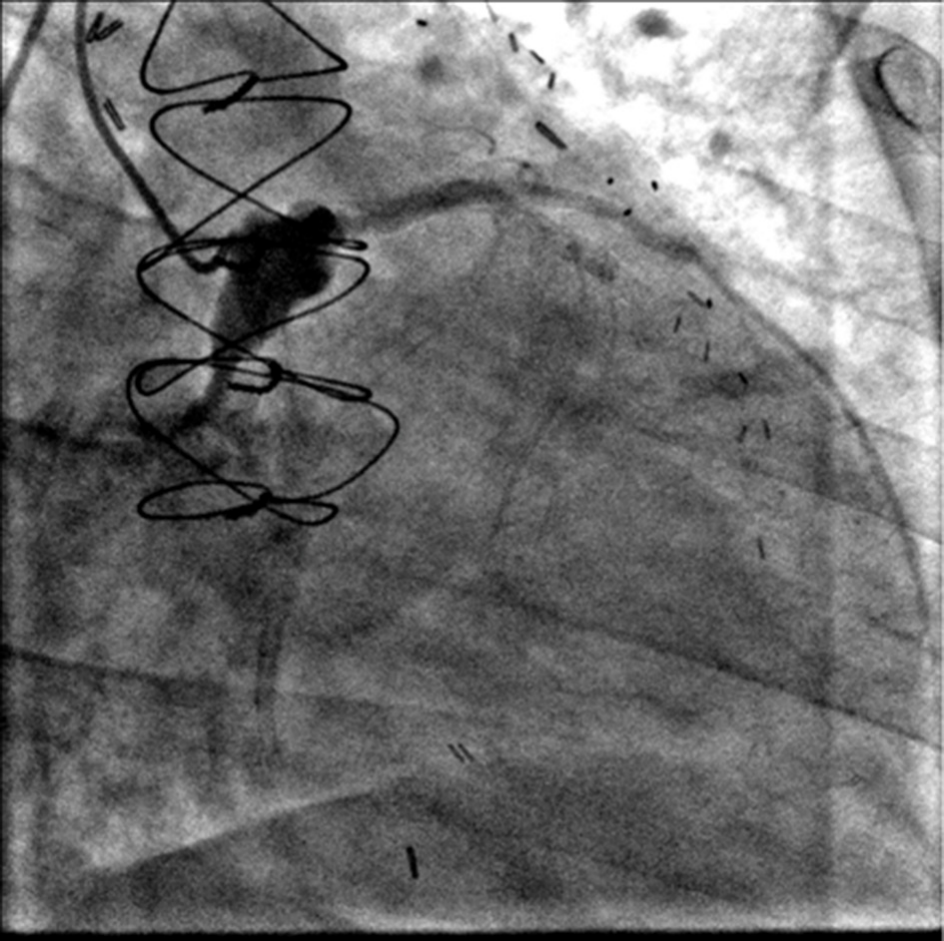
Fistulous connection from the left mid-circumflex coronary artery to the mitral annulus and occlusion of the circumflex distal to this fistula.

Coronary angiography revealed a fistulous connection (see Fig. 1) from the left mid-circumflex coronary artery to the left atrium and occlusion of the circumflex distal to this fistula. Only mild mitral valve regurgitation was present. The coronary angiogram contrast pattern was most consistent with endarteritis due to coronary stent infection with fistulous left circumflex to left atrium connection. Since this was a left coronary artery to left atrium fistula, and the myocardium distal to the fistula origin was mostly infarcted, it was felt that the fistula itself has a negligible haemodynamic impact.

## Treatment

Treatment consisted of vancomycin, rifampin and gentamicin for an 8 week course. Cardiothoracic surgeons determined that the patient was not a surgical candidate due to the infection’s extent and location, which precluded thorough debridement and appropriate reconstruction. Further, surgery was unlikely to reverse myocardium that had already infarcted. Nineteen days after the patient's first positive blood cultures, his cultures finally cleared. Over these 19 days, he had 26 positive blood cultures out of a total of 36 bottles sent. After completion of triple therapy, the patient was placed on daily trimethoprim/sulfamethoxazole (80–400 mg) as lifelong suppressive therapy.

## Outcome and follow-up

The patient had no recurrence of MRSA bacteraemia. After spending over a month in the hospital on the medical floor, he was discharged to a rehabilitation facility. Coronary angiography performed 6 months after diagnosis of his stent infection revealed the continued presence of a large left circumflex to left atrium fistula and an occluded left circumflex artery with a large infarct territory. The patient suffered a fatal cardiac arrest 6 months after his coronary stent infection was diagnosed.

## Discussion

Despite the ubiquity of coronary stents for the management of CAD, a literature review published in 2012 identified only 17 reported cases of published coronary artery stent infections [2]. Diagnosis of coronary stent infections can be elusive, however, so reported rates may be lower than their true incidence [3]. Definitive diagnosis of coronary stent infection requires histopathological evidence of infection, including demonstration of abscess, inflammatory mass, or aneurysm or pseudoaneurysm [4]. In the absence of histological data, a diagnosis of coronary stent infection should be strongly considered in patients who have undergone stent placement within the past 4 weeks [5] – especially patients on whom multiple procedures are performed through the same arterial sheath – and who present with evidence of infection (bacteraemia, significant fever or leucocytosis with no identifiable cause), acute coronary syndrome or an abnormality on cardiac imaging (echocardiography, angiography, computed tomography or cardiac magnetic resonance) [3, 4].

While most published episodes of coronary stent infection occur within 4 weeks of stent placement, infection occurring a year after coronary stent placement has been reported [3]. Select characteristics of coronary stent procedures have been associated with an increased risk for infection, including vascular injury, persistence of an arterial sheath for greater than 24 h [6], repeated punctures of the arterial access site, lengthy procedures and infection at a distal site that could be a potential source of coincident bacteraemia [3]. It is thought that drug-eluting stents pose a high infection risk as compared to bare metal stents, due to their immunomodulatory properties [7]. However, with such low numbers of reported infection, it is hard to definitively identify risk factors associated with stent infections.

The high mortality associated with coronary stent infections can be attributed at least in part to the fact that initial signs and symptoms are non-specific, and bacteraemia, when detected, can be erroneously attributed to another cause. As a result, diagnosis of coronary stent infections is often delayed until a potentially fatal complication arises, including pericardial tamponade, pericardial empyema, coronary artery aneurysm, coronary artery rupture, ventricular rupture, valvular root abscess, endocarditis, coronary artery occlusion and cardiac arrest [8]. The predominant organism associated with stent infections is *S. aureus*, and *Pseudomonas aeruginosa* is the second most commonly reported pathogen [7].

The use of perioperative antimicrobials should be considered for angiographical procedures during which an arterial sheath is predicted to dwell for longer than 24 h or there is repeated puncture of the same vessel during a short time period, and the targeted use of perioperative antimicrobials may reduce the likelihood of coronary stent infection. The optimal antibiotic treatment regimen for established coronary stent infection is not known [7]. Published guidelines exist for the management of prosthetic valve endocarditis therapy, and it is not unreasonable to extrapolate these recommendations to coronary stent infections. For *S. aureus* prosthetic valve endocarditis, which is associated with high morbidity and mortality, Infectious Diseases Society of America guidelines recommend combination therapy, based on evidence demonstrating the superiority of this approach [9]. Infections that present within 10 days of stent placement are potentially amenable to medical therapy alone; for all others, surgical intervention is indicated [2]. The largest review to date cites a 75 % mortality in patients treated with medical therapy alone, as compared to a 33 % mortality in those treated both medically and surgically [2]. However, not all patients are surgical candidates, because procedures to remove all infected hardware and provide new bypass grafts to the heart can be challenging, if not impossible, as was the case with our patient.

This case highlights the diagnostic and therapeutic challenges produced by coronary stent infections, and reinforces the notion that bacteraemia in the setting of recent stent placement, especially when it is complicated by chest pain, should prompt consideration of stent infection. Judicious use of coronary stents and administration of perioperative antibiotics in settings where angiography is accompanied by prolonged sheath dwell or repeated punctures may reduce the incidence of coronary stent infections, and earlier diagnosis of coronary stent infections may prevent the occurrence of complications.
